# The effect of exercise-intensity on skeletal muscle stress kinase and insulin protein signaling

**DOI:** 10.1371/journal.pone.0171613

**Published:** 2017-02-09

**Authors:** Lewan Parker, Adam Trewin, Itamar Levinger, Christopher S. Shaw, Nigel K. Stepto

**Affiliations:** 1 Institute of Sport, Exercise and Active Living (ISEAL), College of Sport and Exercise Science, Victoria University, Melbourne, Australia; 2 Institute for Physical Activity and Nutrition, School of Exercise and Nutrition Sciences, Deakin University, Geelong, Australia; 3 Monash Centre for Health Research and Implementation (MCHRI), School of Public Health and Preventative Medicine, Monash University and Monash Health, Clayton, Australia; University of Birmingham, UNITED KINGDOM

## Abstract

**Background:**

Stress and mitogen activated protein kinase (SAPK) signaling play an important role in glucose homeostasis and the physiological adaptation to exercise. However, the effects of acute high-intensity interval exercise (HIIE) and sprint interval exercise (SIE) on activation of these signaling pathways are unclear.

**Methods:**

Eight young and recreationally active adults performed a single cycling session of HIIE (5 x 4 minutes at 75% W_max_), SIE (4 x 30 second Wingate sprints), and continuous moderate-intensity exercise work-matched to HIIE (CMIE; 30 minutes at 50% of W_max_), separated by a minimum of 1 week. Skeletal muscle SAPK and insulin protein signaling were measured immediately, and 3 hours after exercise.

**Results:**

SIE elicited greater skeletal muscle NF-κB p65 phosphorylation immediately after exercise (SIE: ~40%; HIIE: ~4%; CMIE; ~13%; p < 0.05) compared to HIIE and CMIE. AS160^Ser588^ phosphorylation decreased immediately after HIIE (~-27%; p < 0.05), and decreased to the greatest extent immediately after SIE (~-60%; p < 0.05). Skeletal muscle JNK (~42%; p < 0.05) and p38 MAPK (~171%; p < 0.05) phosphorylation increased, and skeletal muscle Akt^Ser473^ phosphorylation (~-32%; p < 0.05) decreased, to a similar extent immediately after all exercise protocols. AS160^Ser588^ phosphorylation was similar to baseline three hours after SIE (~-12%; p > 0.05), remained lower 3 hours after HIIE (~-34%; p < 0.05), and decreased 3 hours after CMIE (~-33%; p < 0.05).

**Conclusion:**

Despite consisting of less total work than CMIE and HIIE, SIE proved to be an effective stimulus for the activation of stress protein kinase signaling pathways linked to exercise-mediated adaptation of skeletal muscle. Furthermore, post-exercise AS160^Ser588^ phosphorylation decreased in an exercise-intensity and post-exercise time-course dependent manner.

## Introduction

High-intensity interval-exercise (HIIE) and sprint-interval exercise (SIE) are reported to elicit comparable, and in some cases, greater improvements in measures of glycemic control, oxidative stress, and mitochondrial biogenesis, compared to continuous moderate-intensity exercise (CMIE) [1–5]. The mechanisms for improved skeletal muscle adaptation after HIIE and SIE are unclear, but may involve exercise-induced stress protein kinase signaling [6–9].

Physical inactivity and excess adipose tissue can lead to the sustained activation of mitogen and stress-activated protein kinases (SAPK), in-part through increased mitochondrial electron leak and the subsequent production of reactive oxygen species (ROS) [10, 11]. Important ROS sensitive SAPK proteins include c-Jun N-terminal kinases (JNK), p38 mitogen-activated protein kinases (p38 MAPK), and nuclear factor kappa-light-chain-enhancer of activated B cells (NF-κB). Sustained activation of these protein signaling pathways leads to impaired insulin sensitivity in part through serine phosphorylation of the insulin receptor substrate 1 (IRS-1), IRS-1 degradation and attenuation of distal insulin signaling proteins such as Akt substrate 160 (AS160) [11–13]. Paradoxically, acute exercise also results in increased ROS production [14], albeit transiently and predominantly through NADPH oxidase superoxide anion production [15], which is reported to contribute to the transient activation of SAPK signaling in skeletal muscle [16]. In contrast to the sustained activation of SAPK signaling, the transient activation following acute exercise coincides with greater AS160 phosphorylation post-exercise and improved insulin sensitivity [17–19]. Furthermore, exercise-induced SAPK signaling is also linked to the activation of skeletal muscle transcription factors and coactivators that lead to skeletal muscle adaptation and long-term improvements in cardiometabolic health [2, 5, 16, 20].

Although HIIE and SIE training are reported to elicit equivalent and in some cases superior exercise-mediated cardiometabolic adaptations when compared to CMIE [1, 2, 5], the effects of acute HIIE and SIE on post-exercise skeletal muscle SAPK signaling are equivocal. For example, greater metabolic fluctuations induced through intermittent exercise are considered to elicit greater post-exercise p38 MAPK phosphorylation [21]. However, previous studies have reported similar exercise-induced p38 MAPK phosphorylation after acute work-matched HIIE, SIE, and continuous exercise [22, 23]. The effects of low-volume SIE, compared to higher-volume HIIE work-matched to continuous exercise of moderate-intensity, on post-exercise skeletal muscle p38 MAPK phosphorylation are unknown. Furthermore, skeletal muscle JNK and NF-κB phosphorylation and post-exercise insulin protein signaling have yet to be explored after acute HIIE and SIE.

We compared the effects of a single session of HIIE, SIE, and CMIE work-matched to the HIIE, on skeletal muscle SAPK and insulin protein signaling. It was hypothesized that SIE and HIIE would elicit greater skeletal muscle SAPK and distal insulin protein signaling.

## Materials and methods

### Participants

Eight recreationally active adults, 6 males and 2 females, volunteered to participate in this randomized cross-over study. Participant characteristics are reported in Table 1. Exclusion criteria for participation included smoking, musculoskeletal or other conditions that prevent daily activity, symptomatic or uncontrolled metabolic or cardiovascular disease, and females taking oral contraception. To minimize the effect of hormonal fluctuations on outcome measures, females were tested in the early follicular phase of the menstrual cycle (2–7 days after the onset of menses). Verbal and written explanations about the study were provided prior to obtaining written informed consent. This study was approved by the Victoria University Human Research Ethics Committee and carried out in accordance with The Code of Ethics of the World Medical Association (Declaration of Helsinki) for experiments involving humans [24].

**Table 1:** Descriptive characteristics of participants.

Variable	N = 8
Participants	6 males and 2 females
Age (years)	25 ± 2
Height (cm)	179.3 ± 2.9
Weight	79.4 ± 2.1
BMI (kg∙m^-2^)	25 ± 1
W_max_ during GXT (W)	327 ± 25
Max heart rate during GXT (BPM)	183 ± 4
VO_2max_ (ml·kg^-1^·min^-1^)	48.4 ± 4.0

Values are mean ± SEM.

**Fig 1:**
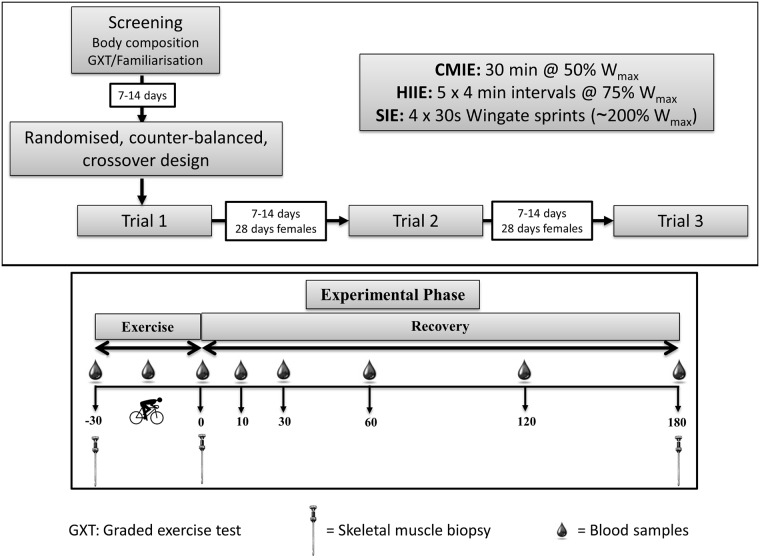
Schematic overview of research methodology. After initial screening and determination of W_max_ and VO_2peak_, participants underwent three exercise sessions, separated by 7–14 days (~28 days for females), in a randomized crossover fashion. Venous blood and skeletal muscle samples were taken at time-points indicated in the figure. CMIE: continuous moderate-intensity exercise. HIIE: high-intensity interval exercise. SIE: sprint interval exercise. GXT: graded exercise test.

Participants were asked to abstain from physical activity (~72 hours), alcohol and caffeine consumption (~24 hours) prior to each trial. Twenty-four hours before their first trial volunteers were asked to consume their habitual diet which was recorded in a diet diary and replicated in their subsequent trials. Participants completed a screening session prior to completing the three different exercise protocols in a randomized crossover fashion, separated by a minimum of 1 week for males and ~4 weeks for females (Fig 1).

### Screening and preliminary testing

Participants were screened via a medical history and risk assessment questionnaire. Eligible participants underwent anthropometric measurement (height and weight) and completed a graded exercise test (GXT) on a cycle ergometer (Velotron, USA) to measure peak aerobic capacity (VO_2peak_) and maximal power output (W_max_). The GXT protocol consisted of 1-minute cycling stages at 50 watts which increased by 25 watts every minute until participants were unable to maintain a cycling cadence of 60 RPM or greater. Expired gases were collected and analyzed via an indirect calorimetry system (Moxus Modular VO_2_ System, USA). The W_max_ obtained during the GXT was used to calculate the workload for the three exercise protocols.

### Experimental phase

On three separate occasions participants reported to the laboratory in the morning after an overnight fast. A resting muscle biopsy and venous blood sample were taken prior to participants undergoing their randomized exercise protocol (SIE, HIIE or CMIE). Immediately following the acute session of exercise, a muscle biopsy and venous blood sample were taken, and participants rested on a bed for three hours. A third muscle biopsy was taken 3 hours after exercise and venous blood samples were taken in the middle of the exercise session, immediately after exercise, and 10 minutes, 30 minutes, 1 hour, 2 hours and 3 hours after exercise.

### Exercise protocols

All exercise sessions were performed on a Velotron cycle ergometer. The SIE protocol consisted of 4 x 30 second all-out (Wingate) cycling sprints, interspersed with 4.5-minute passive recovery periods. Pedaling resistance for the SIE was determined as a torque factor relative to body mass which was optimized during the familiarization session. The HIIE protocol consisted of 5 x 4-minute cycling bouts at 75% of W_max_ (~77% of VO_2peak_), interspersed with 1-minute passive recovery periods. The CMIE protocol consisted of continuous cycling for 30 minutes at 50% of W_max_ (~54% of VO_2peak_), equating to the same total work performed (294 ± 23 kJ) in the HIIE protocol.

### Skeletal muscle and blood sampling

Muscle samples were obtained from the vastus lateralis under local anesthesia (Xylocaine 1%, Astra Zeneca, Australia) utilizing a Bergström needle with suction [25]. The samples were immediately frozen in liquid nitrogen and stored at -80°C until analysis. Venous blood was collected from an antecubital vein via an intravenous cannula and analyzed immediately for blood glucose and lactate using an automated analysis system (YSI 2300 STAT Plus^™^ Glucose & Lactate Analyzer).

### Skeletal muscle protein analysis

To avoid the potential loss of total cellular protein that can occur with centrifugation [26, 27], phosphorylation and abundance of specific proteins in whole muscle lysate were determined with all constituents present (i.e. no centrifugation). Whole muscle lysate was analyzed as previously reported [19]. In brief, thirty cryosections of skeletal muscle (20 μm) were homogenized in buffer (0.125M TRIS-HCL [pH 6.8], 4% SDS, 10% Glycerol, 10mM EGTA, 0.1M DTT, and with 0.1% v/v protease and phosphatase inhibitor cocktail [#P8340 and #P5726, Sigma Aldrich]). Total protein content of muscle lysate was determined using the commercially available Red 660 Protein Assay kit with SDS neutralizer as per the manufacturer’s instructions (Red 660, G-Biosciences, St. Louis, MO, USA). Eight μg of protein was prepared in 3 μl of Bromophenol blue (1%), heated for 5 minutes at 95°C and separated by 7.5% Criterion^™^ TGX^™^ Pre-Cast Gels. The separated proteins were transferred to a polyvinylidene difluoride membrane and blocked with Tris-Buffered Saline-Tween (TBST) and 5% skim milk for 1 hour. Membranes were washed (4 x 5 minutes) with TBST and incubated at 4°C overnight with the following primary antibodies: phospho-SAPK/JNK (Thr183/Tyr185; CST #9251), SAPK/JNK (CST #9252), phospho-p38 MAPK (Thr180/Tyr182; CST #9211), p38 MAPK (CST #9212), phospho-NF-κB p65 (Ser536; CST #3033), NF-κB p65 (CST #8242), IκBα (CST #4814), phospho-IRS-1 (Ser307 in human; CST #2384), phospho-AS160 (Ser588; CST #8730), AS160 (CST #2447), phospho-Akt (Ser473; CST #9271), Akt (#9272), and IRS-1 (Millipore, 06–248). After incubation, membranes were washed with TBST and incubated for 1 hour at room temperature with appropriate dilutions of horseradish peroxidase conjugated secondary antibody. Membranes were re-washed and incubated in SuperSignal West Femto Maximum Sensitivity substrate for 5 minutes prior to imaging. After imaging, membranes were stained via a modified Coomassie staining protocol [19]. All densitometry values are expressed relative to a pooled internal standard and normalized to the total protein content of each lane obtained from the modified Coomassie staining protocol. Where appropriate, phosphorylated proteins are expressed relative to specific total protein content.

### Statistical analysis

Data were checked for normality and analyzed using Predictive Analytics Software (PASW v20, SPSS Inc., Chicago, WI, USA). Comparisons of multiple means were examined using a repeated measures analysis of variance (exercise protocol x time point). Post hoc analysis of significant interaction and main effects were performed using Fisher’s protected LSD test. All data are reported as mean ± standard error of mean (SEM) and statistical analysis conducted at the 95% level of significance (p≤0.05). Trends were reported when p-values were greater than 0.05 and less than 0.1.

## Results

### Blood glucose and lactate

**Fig 2:**
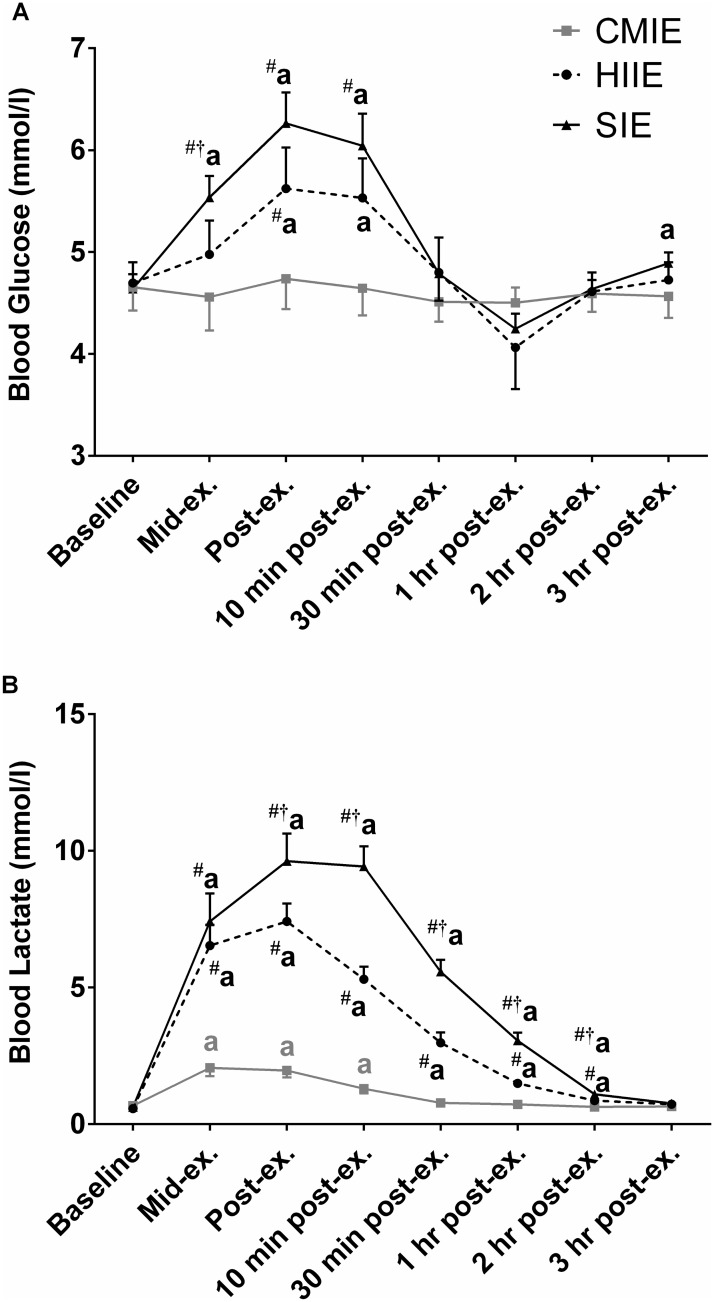
Blood lactate and blood glucose during and after exercise. **(A)** Blood lactate and **(B)** blood glucose response to high-intensity interval exercise **(HIIE)**, sprint-interval exercise (SIE), and continuous moderate-intensity exercise **(CMIE)**. a = p < 0.05 compared to baseline. Significantly different (p < 0.05) at equivalent time point vs # = CMIE and † = HIIE

Significant interaction effects (p<0.05) were detected for blood glucose and lactate (p<0.05). Post-hoc analysis revealed that compared to baseline, blood glucose was significantly elevated (p<0.05) after HIIE, and to the greatest extent after SIE (Fig 2). Furthermore, post-hoc analysis revealed that compared to baseline, blood lactate was elevated after CMIE, HIIE, and to the greatest extent after SIE (Fig 2).

### Skeletal muscle SAPK signaling

**Fig 3:**
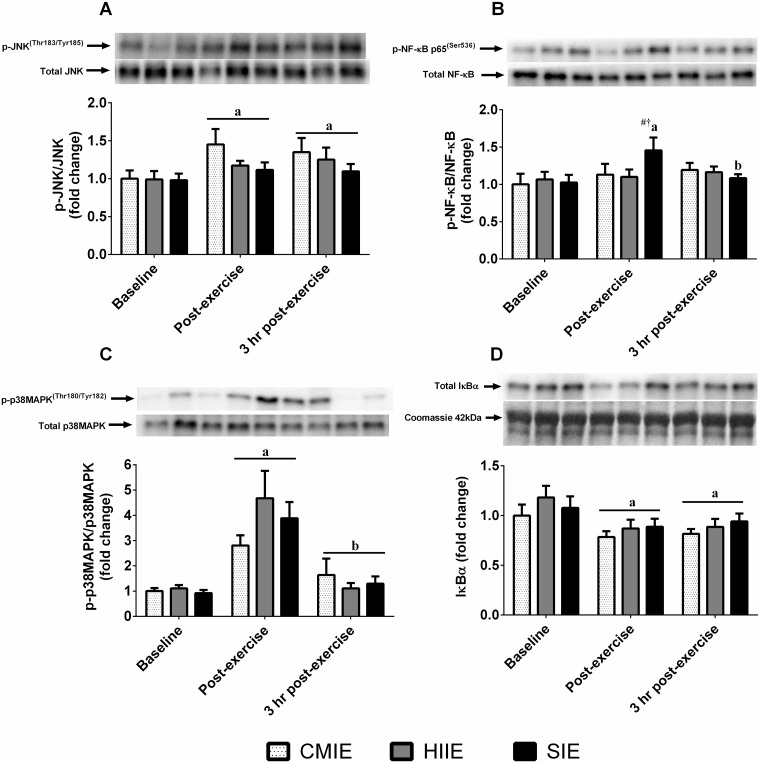
Skeletal muscle SAPK signaling. Skeletal muscle protein phosphorylation relative to total protein content of **(A)** JNK^Thr183/Tyr185^, **(B)** NF-κB p65^Ser536^, **(C)** p38 MAPK^Thr180/Tyr182^, and total protein content of **(D)** IκBα relative to Coomassie protein content, after high-intensity interval exercise **(HIIE)**, sprint interval exercise **(SIE)**, and continuous moderate-intensity exercise **(CMIE)**. a = p < 0.05 compared to baseline; b = p < 0.05 compared to post-exercise. Significantly different (p < 0.05) at equivalent time point vs # = CMIE and † = HIIE.

A significant interaction effect (p < 0.05) was detected for NF-κB p65 phosphorylation. Post-hoc analysis revealed significantly greater (p < 0.05) NF-κB p65 phosphorylation immediately after SIE compared to baseline, and greater phosphorylation immediately after SIE compared to both HIIE and CMIE (Fig 3). Main time effects (p < 0.05) revealed greater phosphorylation of p38 MAPK immediately after exercise, and greater JNK phosphorylation immediately after and 3 hours after exercise compared to baseline (Fig 3). Main time effects (p < 0.05) revealed lower protein abundance of IκBα immediately and 3 hours after exercise compared to baseline (Fig 3).

### Skeletal muscle insulin protein signaling

**Fig 4:**
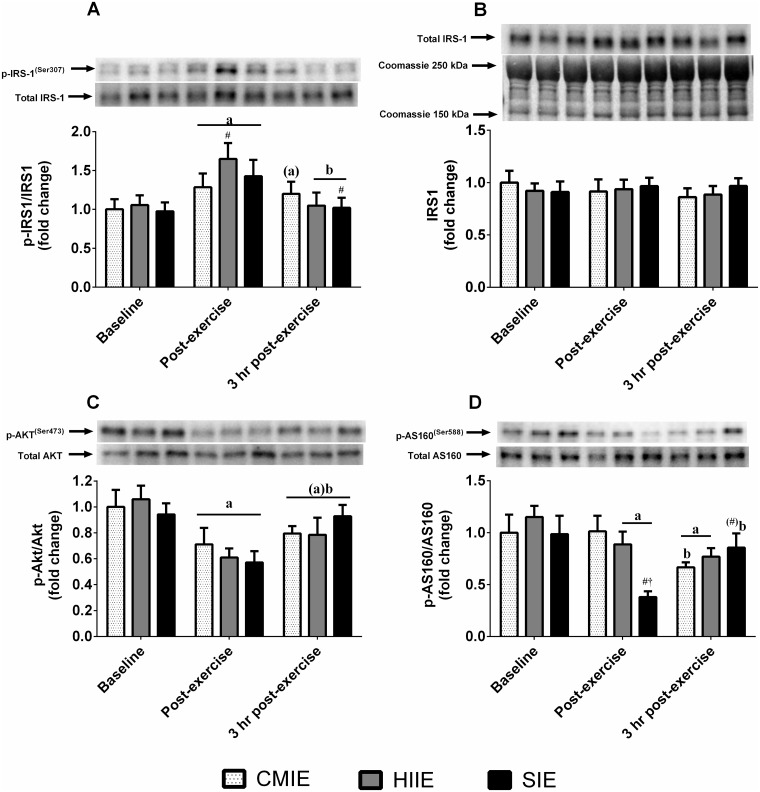
Skeletal muscle insulin protein signaling. Skeletal muscle total IRS-1 content **(B)** and phosphorylation relative to total protein content of **(A)** IRS-1^Ser307^, **(C)** Akt^Ser473^, and **(D)** AS160^Ser588^, after high-intensity interval exercise **(HIIE)**, sprint interval exercise **(SIE)**, and continuous moderate-intensity exercise **(CMIE)**. a = p < 0.05 and (a) p<0.1 compared to baseline; b = p < 0.05 and (b) p < 0.1 compared to post-exercise. Significantly different (p < 0.05) or trend (p < 0.1 in parenthesis) at equivalent time point vs # = CMIE and † = HIIE.

A significant interaction effect (p < 0.05) was detected for IRS-1^Ser307^ phosphorylation. Post-hoc analysis revealed significantly greater IRS-1^Ser307^ phosphorylation immediately after all exercise bouts, and there was a trend for this to remain elevated at 3 hours after CMIE only (Fig 4). IRS-1^Ser307^ phosphorylation was significantly greater immediately after HIIE compared to CMIE, and greater 3 hours after CMIE compared to SIE. A significant interaction effect (p < 0.001) was detected for AS160^Ser588^ phosphorylation. Post-hoc analysis revealed lower phosphorylation of AS160^Ser588^ immediately after SIE and HIIE compared to baseline, and 3 hours after CMIE and HIIE compared to baseline (Fig 4). AS160^Ser588^ phosphorylation was lower immediately after SIE compared to HIIE and CMIE, and was higher 3 hours after SIE compared to CMIE. Phosphorylation of Akt^Ser473^ was lower immediately after exercise compared to baseline and tended to remain lower 3 hours after exercise (Fig 4). Despite increased IRS-1^Ser307^ phosphorylation, total IRS-1 protein was not significantly influenced by exercise (Fig 4).

## Discussion

We report that a single session of SIE elicited greater skeletal muscle NF-κB p65 phosphorylation compared to HIIE and CMIE, a similar increase in JNK and p38 MAPK phosphorylation, and a similar decrease in skeletal muscle IκBα protein content. Thus, despite consisting of less total work than CMIE and HIIE, SIE proved to be an effective stimulus for the activation of stress protein kinase signaling pathways linked to exercise-mediated adaptation of skeletal muscle.

### Exercise intensity and skeletal muscle SAPK signaling

NF-κB p65 phosphorylation in human skeletal muscle was increased immediately after SIE, but not after CMIE or HIIE. It is unclear why NF-κB p65 phosphorylation was not increased after CMIE or HIIE, as NF-κB activity/phosphorylation is increased in skeletal muscle of rodents after 1 hour of swimming and treadmill exercise [7, 28]. It is possible that only intense supramaximal exercise provides sufficient stimulus to increase NF-κB p65 phosphorylation in human skeletal muscle immediately after exercise. In support, Petersen *et al*. [29] reported no change in human skeletal muscle NF-κB p65 phosphorylation immediately after 45-minutes of continuous cycling (71% VO_2peak_) or after cycling to exhaustion (92% VO_2peak_). In addition to NF-κB p65 phosphorylation, transcriptional activity of NF-κB requires ubiquitin-dependent IκBα protein degradation, a process which permits inactive cytosolic NF-κB to translocate to the nucleus [30, 31]. Our findings align with others reporting decreased IκBα protein abundance in skeletal muscle after acute exercise [19, 29, 32]. This decrease appears to occur independent of NF-κB p65 phosphorylation and exercise-intensity. It is possible that our biopsy sampling times may not have captured peak NF-κB phosphorylation with CMIE and HIIE, which is increased one hour after HIIE in human skeletal muscle [19], and is reported to peak 1–2 hours after exercise in human PBMC [31] and rat skeletal muscle [33].

Attenuation of the exercise-induced skeletal muscle NF-κB p65 signaling response in humans and rodents, via allopurinol, apocynin, or n-acetylcysteine treatment/ingestion, coincides with attenuation of PGC-1α, manganese superoxide dismutase, glutathione peroxidase, citrate synthase, and mitochondrial transcription factor A gene expression [6, 7, 29]. As such, greater NF-κB p65 phosphorylation after acute SIE may contribute to the equivalent or superior skeletal muscle and cardiometabolic adaptations previously reported with SIE training [1].

The p38 MAPK and JNK signaling pathways play an important role in exercise-mediated mitochondrial biogenesis and antioxidant defense upregulation [34–38]. We provide evidence that JNK and p38 MAPK phosphorylation are increased to a similar extent after SIE, CMIE, and HIIE work-matched to CMIE. These findings support previous reports of similar post-exercise p38 MAPK phosphorylation after continuous exercise work-matched to high-intensity continuous cycling [39], HIIE [22], and SIE [23]. Furthermore, we showed that exercise-induced skeletal muscle JNK phosphorylation in humans does not appear to occur in an exercise-intensity and/or volume manner, contradicting previous reports in rodents [40, 41]. Recently, Combes *et al*. [21] reported greater phosphorylation of p38 MAPK in human skeletal muscle with intermittent cycling (30 x 1-min intervals at 70% VO2_peak_; 1-minute recovery periods) compared to work and intensity matched continuous cycling (30 minutes at 70% VO_2peak_). It was proposed that increased oscillations of the cytosolic NADH/NAD+ redox state [42] elicited through intermittent exercise may play a larger role in p38 MAPK signaling compared to the manipulation of exercise volume or intensity. It is possible that the metabolic demands induced through HIIE and SIE in this and other studies were insufficient to increase p38 MAPK, and potentially JNK phosphorylation, above that of continuous exercise [22, 23]. Further research is required to confirm these findings with exercise protocols that incorporate greater metabolic disturbances.

The present findings suggest that superior skeletal muscle adaptation previously reported with HIIE and SIE when compared to CMIE [1, 2, 4, 5], may occur through protein signaling pathways independent of p38 MAPK and JNK. Nevertheless, SIE consisted of considerably less total work than HIIE and CMIE, and therefore appears to be an effective exercise mode for stimulating post-exercise skeletal muscle phosphorylation of p38 MAPK, JNK, and in particular NF-κB p65.

### Exercise-intensity and phosphorylation of skeletal muscle insulin protein signaling

We provide evidence that IRS-1^Ser307^ phosphorylation is increased immediately after CMIE and SIE, and to a greater extent after HIIE. Interestingly, IRS-1^Ser307^ phosphorylation was similar to baseline 3 hours after HIIE and SIE. The physiological role of IRS-1^Ser307^ phosphorylation is unclear, as it is reported to both positively and negatively regulate downstream insulin signaling and glucose uptake [19, 43]. Akt^Ser473^ phosphorylation, which is downstream of IRS-1, decreased to a similar extent after all exercise protocols. Surprisingly, further probing of the distal insulin signaling cascade revealed that phosphorylation of AS160^Ser588^ was attenuated in an exercise-intensity and post-exercise time-course dependent manner.

Phosphorylation of AS160 (also known as TBC1D4) results in GTP loading and activation of Rabs, releasing GLUT4 vesicles from intracellular compartments and promoting GLUT4 vesicle plasma membrane docking and glucose uptake [44]. Serine 588 specific phosphorylation of AS160 increases with human skeletal muscle contraction, insulin stimulation via the hyperinsulinaemic-euglycaemic clamp, and may play a role in the acute post-exercise enhancement of insulin sensitivity [17, 19, 45, 46]. Previous research is equivocal, with studies reporting no change [47, 48] or increased phosphorylation of AS160^Ser588^ after exercise in both rodents and humans [17, 19, 49, 50]. We are the first to report decreased AS160^Ser588^ phosphorylation immediately after SIE and HIIE. Using the PAS160 antibody, which primarily detects AS160^Thr642^ but also AS160^Ser588^ [51, 52], Treebak *et al*. [53] also reported a decrease in AS160 phosphorylation immediately after high-intensity continuous cycling exercise (20 minutes, 80% VO_2peak_), whereas phosphorylation was unchanged immediately after CMIE (30 mins, ~67% VO_2peak_). We extend previous findings by reporting that AS160^Ser588^ phosphorylation is similar to baseline 3 hours after SIE, but remains lower after HIIE and CMIE.

The mechanism for the substantial decrease in AS160^Ser588^ phosphorylation immediately after SIE is unclear. The reported elevation in blood glucose during and immediately after SIE, and to a lesser extent after HIIE, suggests a transient counter-regulatory hormonal response previously reported after higher-intensity exercise [54]. Certainly, resistance exercise and extreme muscle damaging exercise inhibit insulin protein signaling [55, 56], likely through mTOR inhibition of the PI3K signaling pathway [57]. However, mTOR signaling does not appear to be activated following acute SIE [9]. Alternatively, excess ROS such as hydrogen peroxide may override the potentiation of insulin signaling through the inactivation of protein tyrosine phosphatases [58–60], by increasing JNK and NF-κB mediated inhibition of the PI3K/Akt signaling pathway [61, 62]. In TNF-α/NF-κB induced insulin resistant human myotubes, targeted interference of the NF-κB signaling pathway restores insulin stimulated AS160 and Akt phosphorylation and glucose uptake, despite minimal effect on JNK phosphorylation [13]. Taken together, it is possible that SIE induced NF-κB signaling may transiently suppress AS160 phosphorylation immediately after exercise. Whether the differential effect of exercise-intensity on post-exercise AS160^(Ser588)^ phosphorylation occurs at other AS160 phosphorylation sites, and whether these changes effect post-exercise insulin sensitivity, are unknown and warrant further investigation.

### Limitations

A potential limitation of the study is a small sample size. However, previous invasive human studies have used similar sample sizes to detect significant changes in SAPK signaling [21, 29, 63]. The combined analysis of both males and females may limit interpretation of the results. Nevertheless, exercise-induced p38 MAPK protein signaling appear to be similar between sexes [64]. Furthermore, in the current study we did not undertake subcellular fractionation, immunohistochemistry, and/or direct measurements of kinase activity due to limited tissue availability. Protein kinase signaling is reported to be spatial-temporally sensitive [30, 65] and as such future studies are required to determine the subcellular localization of protein kinase phosphorylation and kinase activity before and after exercise of different intensities and mode. It is also important to note that the acute activation of protein signaling pathways in skeletal muscle do not always reflect functional changes in protein synthesis and/or adaptations with chronic exercise training [23, 66]. Finally, findings in this study are delimited to young recreationally active adults, the specific exercise-protocols investigated, and the investigation of a single session of exercise. Future research is required to confirm these findings with subsequent bouts of exercise over a longer period of time, in more diverse populations with different exercise protocols.

## Conclusions

These findings demonstrate that p38 MAPK and JNK phosphorylation increase to a similar extent after CMIE, HIIE and SIE. On the other hand, skeletal muscle NF-κB phosphorylation was more responsive to intense exercise. Whether greater NF-κB phosphorylation post-SIE contributes to the previously reported superior benefits of SIE on skeletal muscle adaption warrants further investigation. Surprisingly, only CMIE and HIIE elicited a decrease in phosphorylation of the downstream glucose uptake signaling protein AS160 three hours after exercise, despite substantially lower AS160 phosphorylation immediately after SIE. These findings indicate that the time course of post-exercise AS160 phosphorylation, an important regulator of contraction and insulin-stimulated glucose uptake, is influenced in an exercise-intensity dependent manner. Taken together, exercise-intensity plays a role in regulating the complex SAPK signaling pathways which are known to be involved in the adaptive cardiometabolic responses to exercise.
